# Simulation study of autoregulation responses of peripheral circulation to systemic pulsatility

**DOI:** 10.1186/1753-4631-3-7

**Published:** 2009-07-24

**Authors:** Federico Aletti, Ettore Lanzarone, Maria Laura Costantino, Giuseppe Baselli

**Affiliations:** 1 Dipartimento di Bioingegneria, Politecnico di Milano, P.za Leonardo da Vinci, 32, 20133, Milan, Italy; 2 Laboratorio di Meccanica delle Strutture Biologiche, Dipartimento di Ingegneria Strutturale, Politecnico di Milano, P.za Leonardo da Vinci, 32, 20133, Milan, Italy

## Abstract

**Background:**

This simulation study investigated potential modulations of total peripheral resistance (TPR), due to distributed peripheral vascular activity, by means of a lumped model of the arterial tree and a non linear model of microcirculation, inclusive of local controls of blood flow and tissue-capillary fluid exchange.

**Results:**

Numerical simulations of circulation were carried out to compute TPR under different conditions of blood flow pulsatility, and to extract the pressure-flow characteristics of the cardiovascular system. Simulations showed that TPR seen by the large arteries was increased in absence of pulsatility, while it decreased with an augmented harmonic content. This is a typically non linear effect due to the contribution of active, non linear autoregulation of the peripheral microvascular beds, which also generated a nonlinear relationship between arterial blood pressure and cardiac output.

**Conclusion:**

This simulation study, though focused on a simple effect attaining TPR modulation due to pulsatility, suggests that non-linear autoregulation mechanisms cannot be overlooked while studying the integrated behavior of the global cardiovascular system, including the arterial tree and the peripheral vascular bed.

## Background

The study of the interactions between the control of hemodynamics in large arteries and in the peripheral districts of the arterial tree is only marginally addressed in the literature. In the investigation of global control of circulation, cardiovascular (CV) variability (CVV) research has mainly focused on heart rate (HR) variability (HRV), baroreflex and sympathetic control of vascular tone [1,2]. Several studies concerning peripheral autoregulation have instead shown that a local activity, independent from neural drives, is able to regulate blood flow to tissues: for instance, modulations of peripheral resistances in response to mechanical and metabolic stimuli have been repeatedly observed and modeled [3-8].

Although some studies have taken local mechanisms into account when discussing the genesis and sustenance of slow arterial pressure waves [9-11], to our knowledge no systematic effort for a comprehensive interpretation of the mutual interactions between global control of systemic circulation and local autoregulation has been proposed yet. It may be ascribed to the experimental limitations affecting the in vivo study of microcirculation and the consequently difficult validation of the hypotheses regarding these phenomena, or to the uncertainty about which CV variables carry information relative to the activity of the peripheries of the arterial tree. Neural and humoral control mechanisms of blood flow and pressure act in parallel on HR, peripheral resistances, arterial compliances, venous return, and heart contractility. Therefore, the unfolding of the specific roles of each control represents a challenging task still largely overlooked. Consequently, our focus was not on the arterial tree nor on the peripheral autoregulation, per se, rather on the integration of a complex system.

It is well known that TPR is a major determinant of ABP variability [12]; hence, in the hypothesis of an active response of the peripheral autoregulation to pressure wave features, a mutual interaction between hemodynamics in large vessels and local control of blood flow follows.

To our knowledge, only two works by Aljuri and Cohen [13,14] tried to identify autoregulation induced components of TPR oscillations and, in doing so, they introduced a critical interpretation of TPR as seen in a classical, Windkessel like context. In such a perspective, TPR is assumed as a constant constitutive parameter of the arterial tree [15,16]. However, TPR results from the summation of all peripheral resistances of the system and should therefore reflect the local modulations, which are distributed in the peripheries of the arterial tree.

In this paper, we adopt a model for the *simulation *of hemodynamics in large vessels and autoregulation of microcirculation, in order to formulate hypotheses about the effect of peripheral responses on systemic hemodynamics, which can be hardly investigated through experiments. In particular, the modulation of TPR by local dynamics was simulated in function of the pulsatility of circulation, identified as a powerful drive for autoregulation, with a special regard to the comparison with non pulsatile circulation, which is the typical clinical condition characterizing heart surgery requiring non pulsatile cardiopulmonary bypass (NP-CPB).

Global, neural control mechanisms were not included in this model because our goal consisted in observing the potential effect of local non linear activity on systemic hemodynamics, which implied to fix the tone of sympathetic outflow and temporarily exclude global control mechanisms, though physiologically important.

## Methods

### Model of the arterial tree and microcirculation

**Figure 1:**
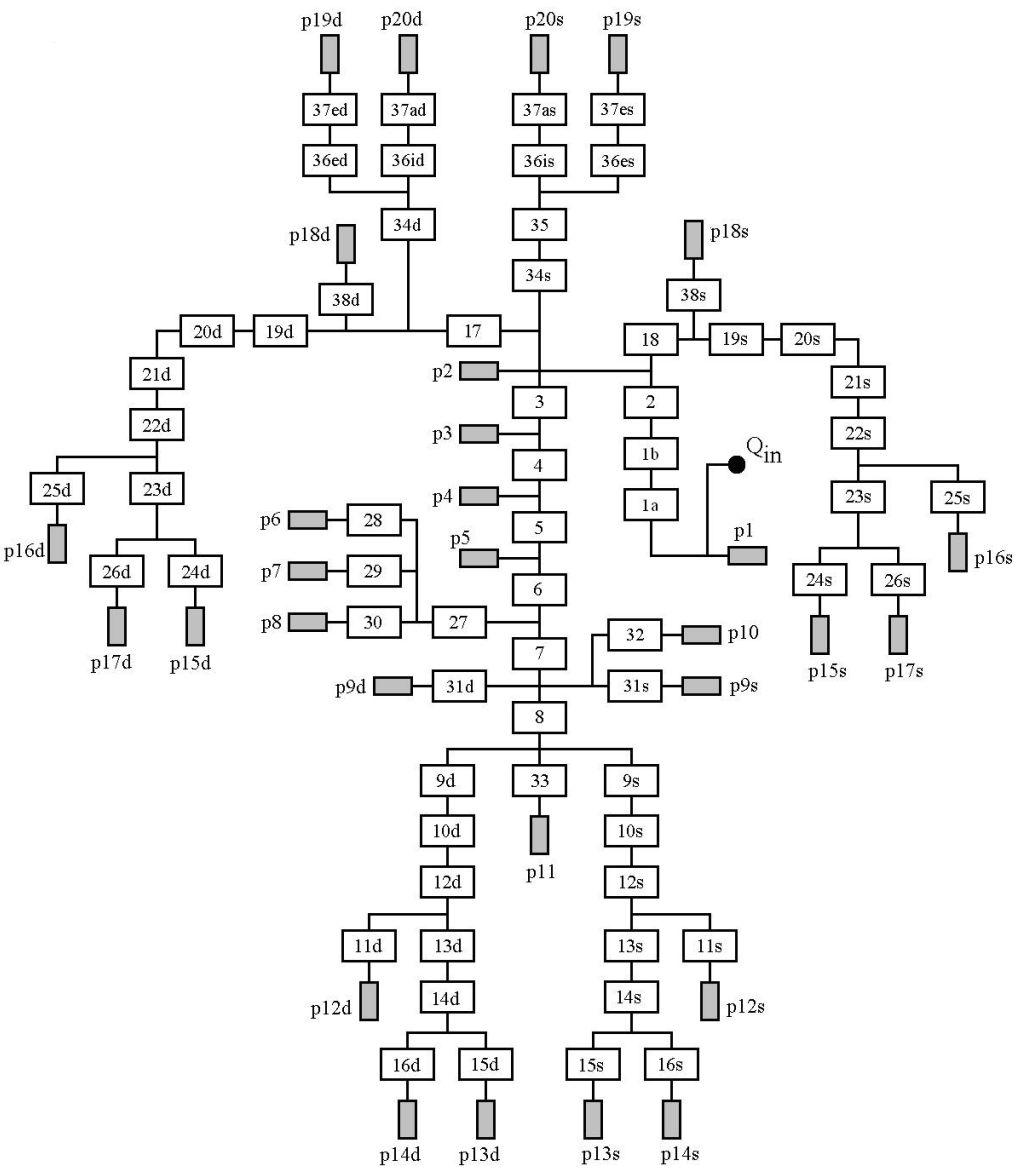
**Lumped parameter model of the arterial tree**. Classical lumped parameter model of the arterial tree [17] (63 segments, white blocks) feeding 30 active, non linear peripheral districts (grey blocks, see fig. 2 for detail).

A lumped parameter model of the arterial tree from the aortic valve to the venous inlet was adopted, according to a previous work [17]. The model consists of a network of 63 RCL circuits in *π *configuration, representing large arteries, and 30 peripheral networks (fig. 1). The system can be solicited by flow or pressure inputs. In this study different heart flow waveforms with a fixed CO and a steady state pressure input were considered.

Each segment of the model was described in terms of ordinary differential equations, representing mass and energy conservation [17].

Peripheral resistance characterization represents the novelty with respect to classical models, since they are not constant resistive elements, as considered either with lumped parameter approaches [15,16] or structured tree approaches [18]. On the contrary, our model includes the analytical description of the passive elastic relaxation of peripheral vessel walls, of the active mechanisms of local regulation of blood flow, and of the filtration of fluids through the capillary walls.

**Figure 2:**
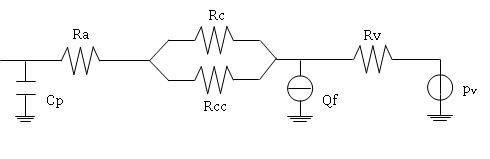
**Model of microcirculation**. Peripheral district of the arterial tree. C_p _is the compliance at the inlet of the peripheral district, R_a _the arteriolar resistance, R_c _and R_cc _form a capillary shunt, R_v _is the venular resistance, Q_f _describes the filtration flow across capillary walls and p_v _sets the venous pressure at 8 mmHg.

The structure of each peripheral network is shown in fig. 2. Each resistance represents the equivalent of a parallel of vessels of the same type (R_a _for arterioles, R_c _and R_cc _for the capillary bed, R_v _for venules) and it is a function of the vessel radius, according to the Hagen-Poiseuille law (eq.1):

(1)

where R is the resistance of the considered element, *μ *the blood dynamic viscosity, l the length of the vessel, r the vessel radius and N the number of vessels in parallel.

The number of arterioles, capillaries and venules varies from network to network while geometrical parameters of single vessels are the same for all districts, in accordance with Lanzarone et al. [17]. Fahareus effect was taken into account for small diameter vessels, according to the *in vivo *formulation of Pries et al [19].

The capacitor C_p _represents the compliance of terminal arteries, which cuts off the high frequencies typical of blood flow in large vessels, in order to obtain low pulsatility, typical of blood circulation in microvessels. Values of the compliances were computed on the basis of the time constant RC of each network [7].

The flow generator Q_f _describes the filtration processes through the capillary wall. The pressure generator p_v _lumps the venous system downstream peripheral circulation and its value was set to 8 mmHg in all the districts.

### Myogenic regulation of pre-capillary resistance

The myogenic regulation controls the arteriolar radius, modulating it in function of the pressure drop on arterioles [3,8,17]. Its action consists in maintaining the flow to a district of the system by regulating the arteriolar tone, and translates into an active regulation characterized by a positive feedback (typical of autoregulation), of vascular tone, lumen and resistance, following changes in pressure. That is to say, a constriction is elicited by a raise in pressure: this raises arteriolar resistance too, causing in turn a further increase in pressure.

The mathematical implementation of this mechanism into the model followed [17], where control equations were based on the experimental work by Davis [3]. The control variable of the system depends on the pressure drop across arterioles. In [17], this pressure was simply the mean pressure inside the arterioles, obtained by averaging the pressures at the inlet and the outlet of the arteriolar resistance. This pressure, here indicated as ppI, was better defined as the difference between the pressure p_c _on the capacity C_p _and half of the pressure drop on arterioles (eq.2):

(2)

where p_c _is the blood pressure at the inlet of the peripheral network, R_a _is the arteriolar resistance and Q_a _is the blood flow entering the peripheral network.

The radius was considered to be a function of ppI. The analytical expressions for the static response of the radius refer to the radius passive component (eq.3), the radius overall behavior (eq.4) and the radius active component (eq.5):

(3)

(4)

(5)

The dynamic response of the radius was described by a first-order dynamics for both the passive and the active component (eq.6,7):

(6)

(7)

A pure delay *θ*_m _was introduced for the active component only, to simulate the time interval between the reading of the arteriolar pressure and the response (eq.8):

(8)

The resulting expression for the radius is the sum of r_pass _and r_act_. All the constants in eqs.3–8 are in accordance with Lanzarone et al. [17].

### Metabolic regulation of post-capillary resistance

The metabolic regulation modulates the peripheral vascular tone evaluating the specific O_2 _consumption by the district and comparing it to the actual oxygen need of the tissue. In case of a deficit of oxygen, the metabolic regulation induces vasodilation, so that the resistance opposed to the flow in the network can be reduced. The implementation of the metabolic control in the model followed [5,17] in attributing its effects only to postcapillary vessels.

Specific venous oxygen concentration C_V_O_2 _in the network triggers the regulation (eq.9):

(9)

where Q_a _is the flow entering the peripheral network through the resistance R_a_, *Cons *the physiological total oxygen consumption of the district, assumed constant in time, and C_A_O_2 _the oxygen concentration in the arterial blood entering the district [17].

A filtered venous oxygen concentration C_r _was introduced by a first order dynamics (eq.10):

(10)

Finally, the radius r_v _response was described by means of a first order dynamics and a pure delay *θ*_v_, starting from C_r _(eq.11,12):

(11)

(12)

where the index I represents the respective reference values, and the function *f *is a saturation function [17]. All the constants in eq.10–12 are in accordance with Lanzarone et al. [17].

The possible role of arteriolar oxygenation [6], though not denied, was not included in this model.

### Fluid balance across capillary walls

The microcirculation model also describes the interactions between the vascular compartment and the interstitial space. The filtration through capillary walls is modeled through a flow generator Q_f _(fig. 2), representing the filtration flow.

The fluid balance between the vascular compartment and the interstitium determines the entity of the fluid exchange. The tissue-capillary fluid exchange is governed by the Starling law of filtration [20]:

(13)

where J is the filtration flow through the capillary wall, and L_p _stands for the permeability index of capillary membranes, Δp(x) is the hydraulic pressure gradient between the blood pressure in capillaries and the interstitial pressure outside microvessels in function of the variable x, which accounts for the length of the microvessel. Δ*π*(x) is the osmotic pressure gradient between the blood pressure in capillaries and the interstitial pressure in function of the variable x.

The capillary radius is subject to the effects of the filtration process through capillary walls [17]. Therefore, this aspect of the analytical model of peripheral districts actually introduces one more controller of the peripheral conductance, specifically of the capillary conductance.

### Simulations: parameters of blood and circulation, inputs to the system, numerical integration

Numerical simulations of circulation were carried out to estimate TPR, to derive the pressure-flow characteristic curve of the arterial tree in presence and absence of local controls of blood flow, and to investigate local dynamics in response to systemic pulsatility, which explain TPR responses.

As regards blood parameters, physiological values were imposed for temperature (37°C), hematocrit (Ht) (45%) and concentration of blood proteins (7.5 g/100 ml of blood). Based on these values, blood density, dynamic viscosity and oncotic pressure were determined according to [17].

The arterial tree was solicited with a set of different flow inputs, imposed at the inlet of the system, in order to investigate the different responses in terms of TPR and to analyze how these systemic differences are due to local activity.

The inputs were either pulsatile flow waves and a non pulsatile one. The system was solicited by different heart flow waveforms at a fixed CO (5 l/min). A steady state pressure input was conversely applied in order to characterize pressure/flow relationships, with and without peripheral control.

**Figure 3:**
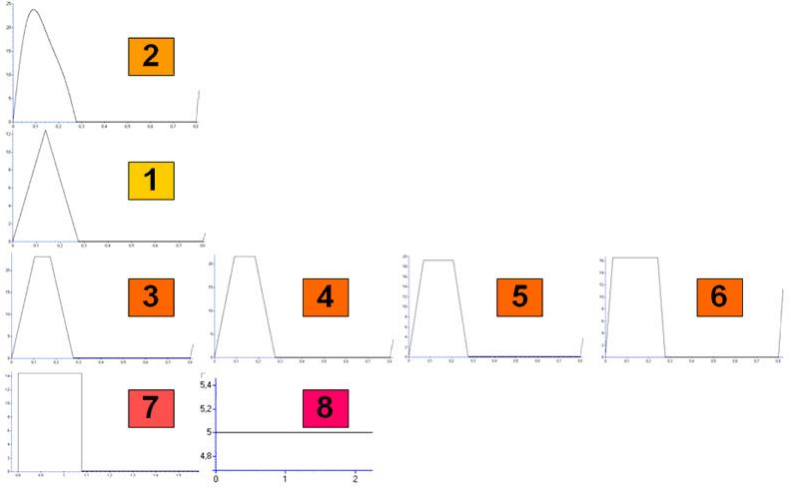
**Inputs to the system**. Inputs to the system: 1) triangular wave; 2) Swanson and Clark's wave; 3–6) trapezoidal waves; 7) rectangular wave; 8) continuous wave.

Pulsatile flow was constrained to zero during diastole, while several different shapes were simulated during systole: in particular, simulations were carried out using the Swanson and Clark wave [21] (mimicking the physiologic cardiac waveform), a triangular wave, four trapezoidal waves, each with a different length of the period of maximum ejection, and a rectangular wave (fig. 3). As regards systole duration, it depends on heart rate (HR) according to Katz-Feil equation [22]:

(14)

where T_s _is the systole duration in s, and HR, which was set at 1.25 Hz (75 bpm).

The idea behind the adoption of this set of input waves was to look for a relation between different degrees of pulsatility and the overall response of the arterial tree, reflected in mean TPR changes.

CO and HR were fixed so that the effect of pulsatility on the system could be evaluated under equal amplitude of stroke volume in any cardiac cycle.

TPR was computed according to the relationship between mean arterial pressure (MAP) and CO, assumed linear and expressed by the hydraulic analogue of the first Ohm's law (eq.15):

(15)

where *p *is blood pressure (MAP considering the entire arterial tree) *Q *stands for flow (equivalently, CO) and *R *is the vessel resistance (TPR). Based upon this equation, TPR can be consequently expressed by their ratio as follows (eq.16):

(16)

MAP was computed as the mean pressure in the segment 1a of the model. A more accurate definition of TPR includes the difference between MAP and central venous pressure (CVP) in the numerator of eq.16. Still, since our model does not describe the venous side of circulation but only pictures the arterial side of circulation, TPR definition did not take CVP into account.

## Results

### TPR of the arterial tree under different cardiac output waveforms

**Figure 4:**
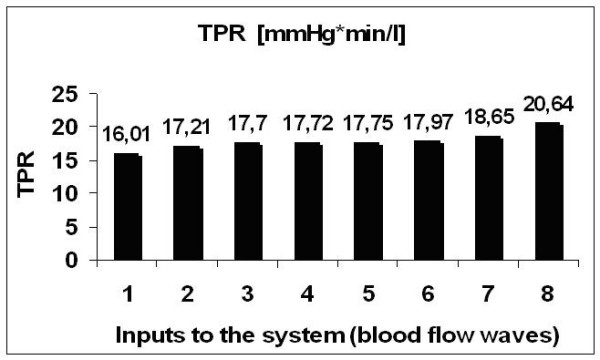
**TPR of the arterial tree in presence of different aortic flow waves**. TPR of the arterial tree (in mmHg.min/l) for different flow waves at the aortic valve. Order of shapes: 1) triangular wave; 2) Swanson and Clark's wave; 3–6) trapezoidal waves; 7) rectangular wave; 8) continuous wave.

Computation of TPR (eq. 16) showed a decrease in TPR with the degree of pulsatility of the input wave, (fig. 4). TPR was lower under a triangular input, it increased under the Swanson and Clark input, it further increased for trapezoidal inputs while the trapezoidal shape tended to a rectangular shape, and it reached the maximum value when the input was represented by a continuous flow. Therefore, the highest value of systemic vascular resistance was obtained in presence of a non pulsatile circulation, while a pulsatile perfusion modality (in particular a physiologic waveform, as mimicked by Swanson and Clark's Wave) was able to keep TPR lower. This provided an indirect, simulated suggestion that local regulation could work better and satisfy its goal of maintaining blood flow to a tissue or organ unchanged, in presence of a pulsatile perfusion modality, rather than under a non pulsatile regime of circulation, as during NP-CPB.

### Pressure-flow characteristic of the arterial tree

**Figure 5:**
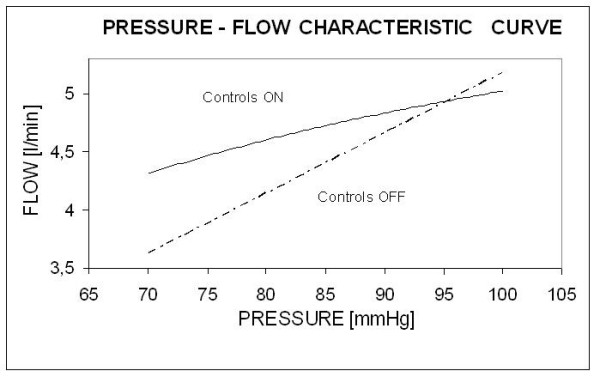
**Pressure-Flow characteristic curve of the arterial tree**. Pressure-Flow characteristic curve of the arterial tree, when local controls are active (solid line, limited slope) and when no local activity is considered (dash-dotted line, proportional flow increase).

The system was solicited by the Swanson and Clark flow waveform (HR = 75 bpm) and steady pressures. CO was computed for values of pressure equal to 70, 75, 80, 85, 90, 95 and 100 mmHg, both in presence and absence of local controls, and two curves ("controls off" and "controls on") were derived interpolating the working points (pressure at the inlet and corresponding CO) and are plotted in fig. 5.

The pressure-flow characteristic curve of the arterial tree was linear in absence of the effect of local controls. This basically implied a constant value of TPR, as typically assumed by classical lumped parameter models, in spite of changes in the steady value of pressure applied at the inlet of the arterial tree. On the other hand, the curve obtained with active local control showed a non linear behavior and lower steepness. The intersection between the two curves represents the physiological working point of the system, where the metabolic demand is balanced by CO.

### Local responses to systemic pulsatility

**Figure 6:**
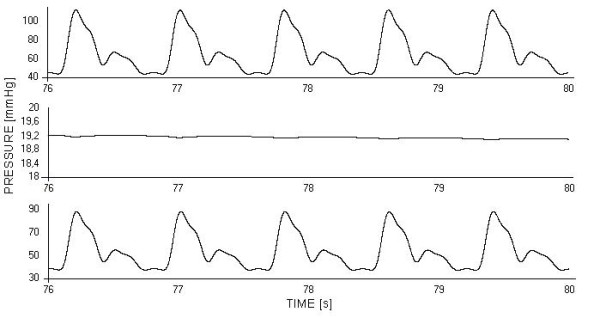
**Pressure time course along microcirculation in the peripheral district p16s**. From top to bottom, the time course of: p_c _(pressure at the inlet of the peripheral district), p_inm _(pressure at the inlet of the capillary bed), ppI (pressure that drives the myogenic control). A generic district is considered. Steady state under Swanson and Clark cardiac flow wave.

The time course of p_c _(pressure at the inlet of the periphery), p_inm _(pressure at the inlet of the capillary bed) and ppI (control variable of the myogenic autoregulation) (eq.2) in a generic district of the upper body was computed after the system reached a steady state when the input was the Swanson and Clark wave, and displayed over few cardiac cycles (fig. 6). This aspect was investigated in order to understand how pressure dynamics evolve along the peripheral network, from its arteriolar inlet to the capillary bed, affecting the control variable of the myogenic regulation of pre-capillary resistances. In table 1, the response of the generic periphery p16s (in terms of mean values of the aforementioned pressures, arteriolar resistance and equivalent resistance of the district) was computed in presence and absence of pulsatility.

**Table 1:** Mean values of pressures and resistances in a peripheral network as a function of pulsatility and controls.

	PULSATILE	NON PULSATILE	Unit
p_c_	68.09	67.33	mmHg
p_inm_	19.08	21.27	mmHg
ppI	56.27	56.08	mmHg
Arteriolar resistance	548.13	556.37	mmHgmin/l
Peripheral resistance	1298.81	1341.70	mmHgmin/l

Parameters of the response of peripheral network p16s in the presence and in the absence of pulsatility (p_c _is the pressure at the inlet of the peripheral district, p_inm _is the pressure at the inlet of the capillary bed, ppI is the pressure which acts as the drive variable of the myogenic regulation).

## Discussion

The sensitivity of TPR to aortic flow shapes and harmonic content (fig. 4) appeared to depend on the superposition of the active peripheral responses in all districts.

In our simulated results, the propagation of pressure waves along the arterial tree and the degree of residual pulsatility at the inlet of peripheries was affected by the flow waveform at the inlet of the arterial tree. As a consequence, a constant TPR, as hypothesized by a linear model, should be a theoretical concept bound to specific conditions. Changes of an apparent TPR are present and supposed to derive from the complex peripheral modulation, which is responsive not only to mean pressure and flow values, but also to other dynamical factors such as the shape of the systemic input waveform. Any type of pulsatile wave was able to induce a lower TPR than without systemic pulsatility.

In other terms, the global system under periodical regime sets around mean values (alias, DC components) which are influenced by the forced oscillations and their harmonic content, which is a typically non-linear behavior.

Translated into the field of NP-CPB, this may support theories and experimental work proposing the use of pulsatile devices in heart-lung machines, in order to reduce and possibly minimize the invasiveness of the procedures. Several studies showed that the presence of a pulsatile flow CPB proves to be beneficial in terms of better district and organ perfusion, compared to the continuous flow CPB [23], although results in this sense are not conclusive and still rather controversial. Recent studies confirmed a different behavior, underlining that the better perfusion can be obtained only in the presence of an adequate reproduction of physiological pulsatility, in terms of left ventricle flow waveform [24,25]. In some cases [25], experimental results have also specifically shown a better preservation of microcirculation, which could be a validating element for our simulated results. However, the problem of vascular responses to a pulsatile or non pulsatile flow CPB, which was also studied from different perspectives with respect to classical assumptions on CV oscillations [26-28], is still widely debated and, even neglecting the potential effects which pulsatility may have on baroceptors stimulation or other global regulatory mechanisms, understanding the response of local activity may shed light on the combined interactions of pulsatility, hemodilution, tissue-capillary fluid exchange and vasomotion.

The pressure-flow characteristic (fig. 5) showed an expected behavior of controls stabilizing flow against mean pressure changes and largely reducing slope, coherently with the modeling description of autoregulation in [29].

This result was also considered a validation for our control equations: as previously pointed out, local controls of blood flow always aim at preserving blood flow to tissues by varying vessels resistance in response to changes in blood pressure. Thus, it appears that autoregulation itself, considering an unchanging sympathetic tone on vessels, may produce modulations of peripheral resistances. Therefore, TPR should not be considered constant as in the hydraulic adaptation of Ohm's law (eq.15), while, from the point of view of autoregulation mechanisms, blood flow is. TPR and ABP modulations mutually affect one each other in virtue of the controls of blood flow and arterial pressure.

Moreover, such a result appeared consistent with experimental observations [30], that demonstrated that autoregulation does modulate regional and systemic vascular resistance in the absence of reflex neurohormonal control of the circulation.

Since our simulations were carried out under the same value of mean CO, the metabolic needs of all peripheral districts were considered matched by their flows. Thus, the myogenic control appeared to be the main responsible for local responses sensitive to flow dynamics, modulating peripheral resistances in response to pressure fluctuations. The time courses of p_c_, p_inm _and ppI (fig. 6) clearly showed that the variable ppI (eq.2), which drives the myogenic regulation, basically had the same dynamic characterization of the arterial pressure at the inlet of the peripheral network because pressure at the inlet of the capillary bed was fundamentally non pulsatile, as an effect of the role of the capacitor C_p_. However, the lumped representation adopted did not permit to exclude residual pulsatility in the initial segment of the capillary bed, according to compliance/resistance distribution.

The simulated response of the myogenic control to systemic pulsatility (table 1) showed a lower arteriolar resistance in order to keep the physiological perfusion of peripheries unaltered: hence, the precapillary pressure drop reduced as well and the working pressures in the whole peripheral district are lower than under a non pulsatile regimen of perfusion. The lower value of resistance obtained in the periphery p16s in presence of pulsatility confirmed that the macroscopic effect shown by TPR in fig. 4 is a reflection of phenomena occurring at the peripheral level.

Summarizing, the core of the discussion of the results of the simulated studies that were presented here can be synthesized in a few key points:

1. Results of our simulations hinted that the myogenic mechanism and the peripheral compliance, which buffers the systemic pulsatility at the inlet of any peripheral network, may represent the link between systemic and peripheral circulation.

2. The combined effect of non linearity due to controls and to Hagen-Poiseuille law (which, although not the object of this discussion, has to be considered as a further element of non linearity because it states that the radius of a vessel and its resistance are related through the inverse fourth power of the radius) results in a lower peripheral resistance under pulsatility.

3. Pulsatility is able to elicit rhythmical oscillations of the arteriolar walls, due to the action of the myogenic mechanisms, whose net effect consist in a decrease of arteriolar resistances.

## Conclusion

The rationale of this study identified pulsatility as a strong drive in peripheral responses. The idea that autoregulation is responsive to cardiac output (CO) dynamic properties [13] and is characterized by a positive feedback response to variations of pressure, could also add material, in a different perspective, to the discussion regarding the concept of "positive feedback" proposed and theorized by Malliani [31], referring to positive feedback autonomic responses, and constituting a distinct contribution to arterial control from negative feedback reflexes.

**Figure 7:**
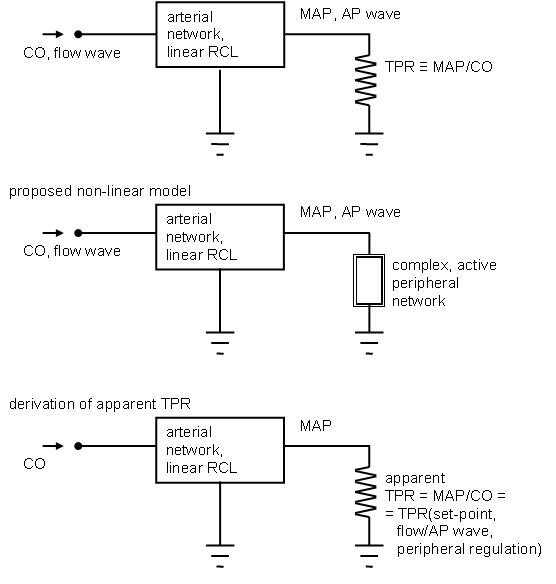
**Schematic approach to the modeling of system-periphery interactions**. From top to bottom, a classical linear modeling approach, our proposed modeling approach, and the consequent derivation of apparent total peripheral resistance (TPR) from the nonlinear modeling of peripheries. Legend: CO stands for cardiac output, AP for arterial pressure, MAP for mean AP.

The concept of "apparent" TPR (explained in fig. 7), reflecting all distributed non linearities of the arterial tree, can be proposed as an integration of the classical Windkessel-like interpretation of TPR.

The presented simulated results may also encourage efforts in experimental microcirculation physiology towards the integration of results deriving from the analysis of isolated microvessels and the investigation of the whole circulatory effects. Both experimental and modeling developments may also cast new light on the contribution of other dynamic components, not addressed here, but which could be easily included in the proposed description of periphery to system interactions. For instance, locally induced vasomotion [32], associated with intracellular Ca^2+ ^modulation [33], may potentially contribute to the modulation of apparent TPR in a non-linear portrait, thus affecting important systemic features such as the heart workload.

Nonetheless, it would be very important to envision possible fields of applications for simulation studies of very significant physiological mechanisms that result deeply affected by relevant pathological conditions or induced altered conditions. In this paper, heart surgery requiring CPB was mentioned, and it certainly represents a major condition for which this type of modeling may prove helpful.

## Competing interests

The authors declare that they have no competing interests.

## Authors' contributions and information

FA implemented the model of microcirculation, carried out the simulation studies, and carried out the discussion about the role of local control on TPR modulations; EL and GB projected the simulation study, contributed to the discussion of the results, and revised the paper; GB conceived the general goals of the work, and proposed the synthesis of the proposed critical interpretation of the Windkessel-derived TPR; MLC proposed the application of the model to the simulation of non pulsatile circulation, and contributed to the interpretation of the respective results. FA is post-doc fellow with the Dipartimento di Bioingegneria, Politecnico di Milano. EL is post-doc fellow at the Politecnico di Milano. MLC is full professor at the Dipartimento di Ingegneria Strutturale, Politecnico di Milano. GB is full professor and chair of the BD and MS programs in Biomedical Engineering, Politecnico di Milano.
